# Supplementary material for: Stacking Model‐Based Classifiers for Dealing With Multiple Sets of Noisy Labels
Source: Biom J. 2025 Mar 12;67(2):e70042. doi: 10.1002/bimj.70042 (PMC11898607; doi:10.1002/bimj.70042)
Supplement: Supplementary file 1 — Supporting Information [file BIMJ-67-e70042-s001.zip › density-based-ensemble-model-main/competitors_real_data/netlab-master/netlab-master/help/rosegrad.htm]

Netlab Reference Manual rosegrad


# rosegrad

## Purpose

Calculate gradient of Rosenbrock's function.

## Synopsis

```
g = rosegrad(x)
```

## Description

`g = rosegrad(x)` computes the gradient of Rosenbrock's function
at each row of `x`, which should have two columns.

## See Also

`demopt1`, `rosen`

---

**Pages:**
Index


---

Copyright (c) Ian T Nabney (1996-9)
